# Diagnostic tool for initial evaluation of the intracranial pressure on computed tomography in pediatric patients with headache

**DOI:** 10.1371/journal.pone.0216812

**Published:** 2019-05-14

**Authors:** Tetiana Bartsikhovsky, Miriam M. Klar, Inessa Bekerman, Saida Nagieva, Sigal Tal

**Affiliations:** 1 Department of Radiology, Shamir Medical Center (Assaf Harofeh), Zeriffin, Israel; 2 Sackler Faculty of Medicine, Tel Aviv University, Ramat Aviv, Israel; Federal University of São Paulo, BRAZIL

## Abstract

**Background:**

Headache is one of the most common complaints among pediatric patients and can be due to many causes, some benign but others potentially seriously. Increased intracranial pressure, which is known to cause papilledema, is a serious cause of headache, and immediate diagnosis is critical, although difficult. The current study evaluates the diagnostic value of optic nerve sheath diameter (ONSD) and eyeball transverse diameter (ETD) ratio in pediatric patients presenting with headache and papilledema.

**Methods:**

A retrospective analysis of all pediatric patients undergoing head computed tomography scans between January 2013 and December 2015. Patients with normal brain scans were included in the study. Patients presenting with headache underwent funduscopic evaluation and grouped as either headache with papilledema or headache without papilledema. A control group of patients without headache was also included. Studies were reviewed blindly by a neuroradiologist and ONSD and ETD for both eyes were measured.

**Results:**

ONSD/ETD index was found to have significantly higher values (p<0.001) in patients with papilledema (median 0.24, interquartile range (IQR) = 0.22–0.25) compared to patients without papilledema (median 0.18, IQR = 0.16–0.19) and the control group (median 0.17, IQR = 0.15–0.18). The ONSD/ETD index showed excellent discrimination ability for patients with headache and papilledema (AUC = 0.96, 95% CI, 0.94–0.99). The ONSD/ETD index of 0.21 was found to have a sensitivity and specificity of 82% and 93%, respectively, for identifying pediatric patients with headache and papilledema.

**Conclusion:**

Our study shows that ONSD/ETD index of 0.21 can be used as an easy-to-use reference tool for diagnosing papilledema and elevated intracranial pressure in pediatric patients.

## Introduction

Headache is one of the most common complaints among pediatric patients, starting as early as early childhood, and the cause of many pediatric visits to emergency departments [1,2]. Prevalence increases with age throughout childhood, reaching 80% in adolescents [3]. While the majority of headache cases are secondary to acute febrile disease, there is a vast differential diagnosis spectrum, including respiratory tract infections, viral meningitis, migraine, tension-type headache, trauma, increased intracranial pressure (ICP), and intracranial pathologies [1, 4]. The clinical implications of these headache causes range from mild and self-resolving to serious and potentially life-threatening. As such, rapid identification of the ‘dangerous’ headaches is crucial when approaching pediatric patients with headaches. Signs and symptoms that raise suspicion for ICP, such as nocturnal headache or starting when waking up, postural changes, ocular palsies, transient visual obscurations, papilledema, nausea, and vomiting are of particular importance for identification of potentially serious headaches [5]. Unfortunately, acute increases in ICP can be difficult to diagnose in pediatric patients due to the fact that the signs and symptoms of increased ICP are often nonspecific [6], although papilledema is believed to be a reliable clinical indicator of elevated ICP and is therefore widely used as a screening tool [7–12]. Hence, pediatric patients with headache who are suspected to have increased ICP require special clinical attention and detailed diagnostic evaluation including ophthalmological evaluation, lumbar puncture, and neuroimaging.

The possibility of ICP assessment by measuring changes in the optic nerve sheath diameter (ONSD) on imaging has been studied for several decades. Because cerebrospinal fluid (CSF) cavities are connected with subarachnoid space underneath the optic nerve, increased ICP is hypothesized to cause the transmission of CSF through these spaces intraorbitally, leading to ONSD distention [13–15]. This increased CSF pressure also impinges on the optic disc, causing disc swelling; this explains why papilledema occurs in the presence of increased ICP and why diagnosing papilledema may be used as a surrogate for diagnosing increased ICP.

Multiple studies have suggested ONSD measurements, which can be obtained through CT, ultrasonography, or MRI as a diagnostic tool for increased ICP in patients with traumatic and non-traumatic brain injury, including intracerebral hemorrhage, idiopathic intracranial hypertension (IIH), meningitis, cardiac arrest, and various other pathological conditions that may‏ lead to ICP elevation, although uncertainty regarding the accuracy of ONSD measurements lead to the investigation of the ratio of ONSD to the eyeball transverse diameter (ETD), ONSD/ETD index, in CT examinations [13,16,17]. Previous studies have demonstrated that this ONSD/ETD index achieves a smaller standard deviation comparing to ONSD measurements alone, making it a much more precise method for increased ICP diagnosis [16]. Unfortunately, most of the studies were performed in adults and to the best of our knowledge similar studies in children have not been published. We therefore aim to examine the correlation between elevated ONSD/ETD index values with the presence of papilledema, the later serving as a clinical equivalent for elevated ICP, in pediatric patients.

## Methods & materials

### Participants

**Fig 1:**
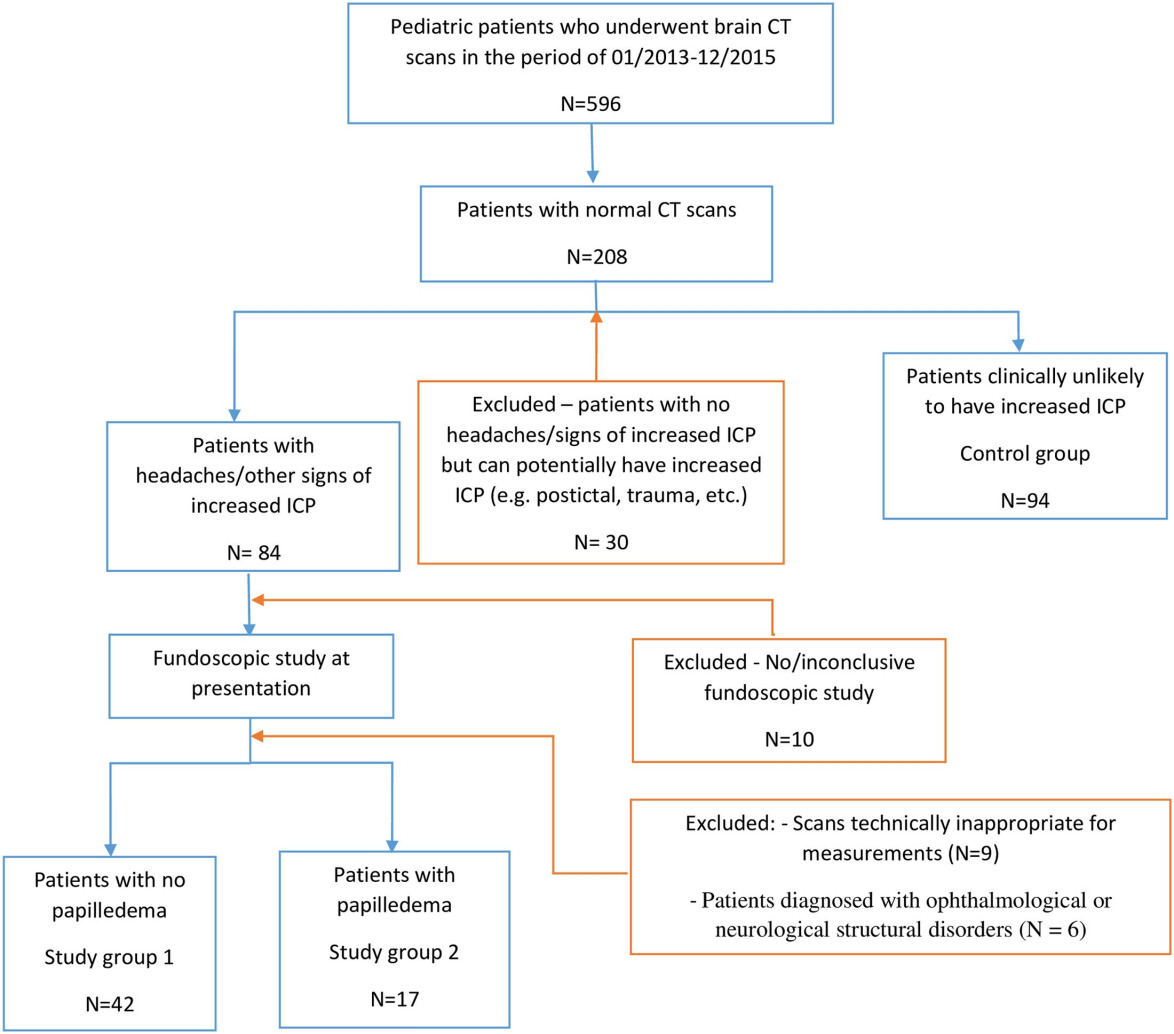
Study population.

This study was conducted at a general secondary level medical care center with specialized pediatric services available. The study was approved by the hospital’s Institutional Review Board (IRB), Assaf Harofeh Medical Center, Zeriffin, Israel. The requirement for informed consent was waived for this retrospective study. The current study retrospectively analyzed 596 consecutive pediatric patients who had a brain CT scan performed at our institution between January 2013 and December 2015. Of these patients, only those with normal scans were included in the study. Patients presenting with a primary complaint of headache and/or other signs of elevated ICP were selected. Most of these patients then underwent funduscopic evaluation in order to diagnose papilledema. The fundoscopic study is a part of routine work up of suspected increased ICP in our institution and is held by ophthalmologist on call. These patients were then stratified into two groups based on presenting symptoms and funduscopic findings: patients without signs of papilledema composed study group 1 and patients with signs of papilledema composed study group 2. Subjects who underwent brain CT studies for various reasons not related to elevated ICP and were unlikely to have papilledema served as the control group. Patients who had no complaints of headache and/or other signs of elevated ICP but potentially could have increased ICP (e.g. postictal, trauma, anoxic-ischemic brain damage) were not included in study. Patients excluded from the study included those presenting with headache and/or signs of elevated ICP but lacking funduscopic evaluation, those with inconclusive funduscopic findings, those diagnosed with ophthalmological or neurological structural disorders of neuro-ophthalmic axis, and those with motion artifacts on brain scan for which appropriate measurements could not be performed. The flow chart with inclusion and exclusion of patients is detailed in Fig 1.

### Data collection

The data was collected using the hospital electronic information system data base and the radiological information system. CT scans and medical records of the study participants were analyzed. Additional documented information included basic demographic information, past medical history, and presenting symptoms.

### Imaging & measurements

All examinations were performed on an MDCT Philips iCT (256 channels) (Philips Healthcare, Cleveland, OH) with section thickness of 3 mm according to routine brain protocol. Scans were reviewed independently by two radiologists (neuroradiologist (I.B.) with more twenty years of experience, radiologist (T.B.) with 3 years of clinical experience) who were blinded to the clinical data. If no cause for elevated ICP was identified on primary interpretation of the CT scans, then ONSD and ETD were measured in both eyes and recorded in millimeters.

**Fig 2:**
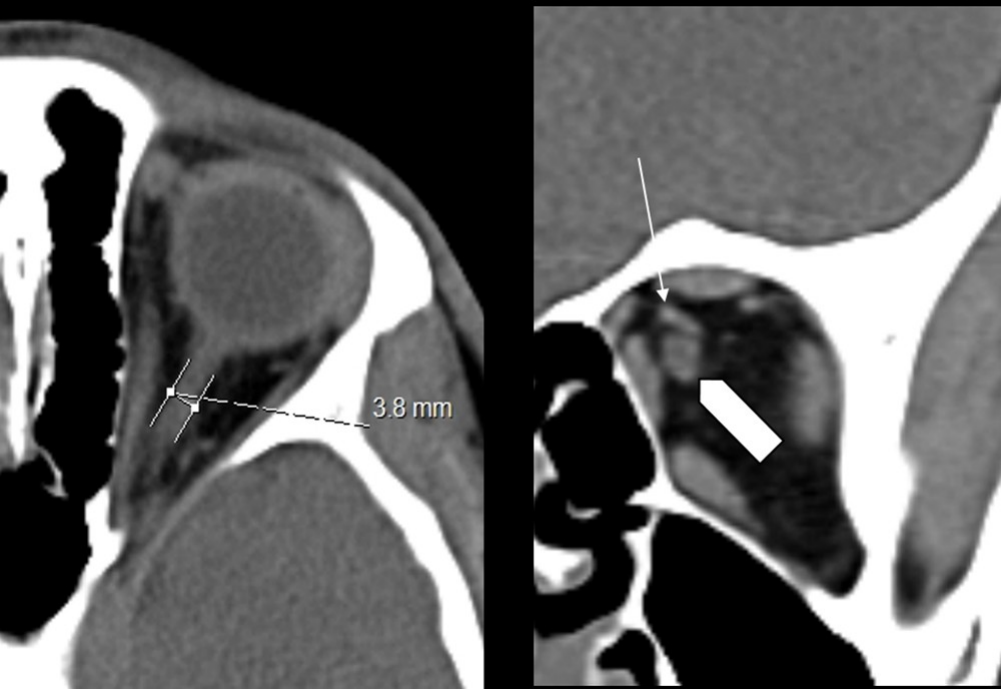
ONSD measurements. A. The measurement of the optic nerve sheath diameter on axial noncontrast CT scan at a point where the ophthalmic vein (thin white arrow) crosses the optic nerve (thick white arrow). B. Coronal images used as reference. The white arrow shows the ophthalmic vein crossing the optic nerve.

**Fig 3:**
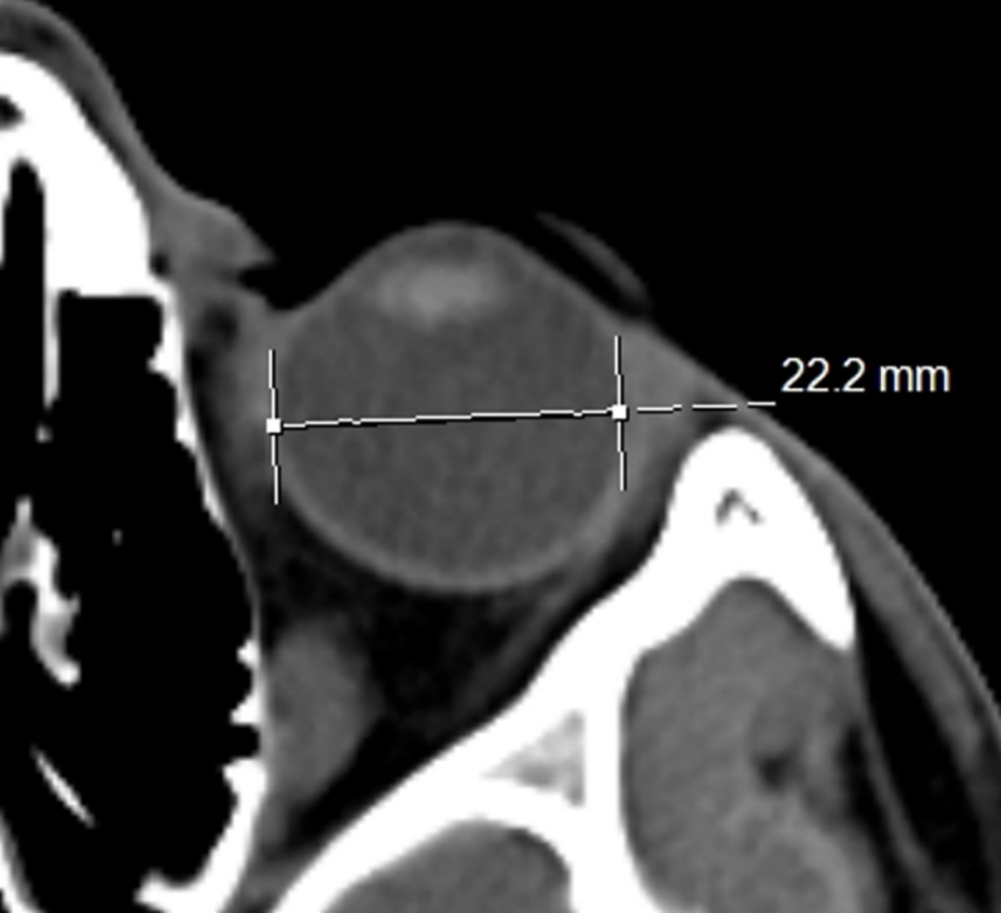
ETD measurement in the same patient as in Fig 1. ETD is measured from retina to retina on axial images. Usually the largest ETD and the ONSD are located in different horizontal planes and are measured on different slices.

Measurements were performed on axial images. Coronal images were used as a localization reference. All structures were measured manually using hand-held digital calipers. Same window parameters (Spine window—WW 60, WL 360) for all measurements were used. ONSD was measured at the point where the ophthalmic vein crosses the optic nerve (Fig 2). Maximal eyeball diameter was measured on the axial slice where the lens appeared the largest to determine ETD (Fig 3). ONSD/ETD ratio was calculated for each patient.

### Statistical analysis

Continuous variables were evaluated for normal distribution using histogram and described using median and interquartile range (IQR). Categorical variables were described using frequency and percentage. Association between continuous variables were assessed using Spearman’s correlation coefficient. Categorical variables were compared between groups issuing Chi-Square tests or Fischer’s Exact test. Continuous variables were compared using Kruskal-Wallis test or Mann-Whitney test. A receiver operating characteristics (ROC) curve was constructed and the area under curve (AUC) was measured and used to evaluate the discrimination ability. Interobserver agreement was evaluated using intraclass correlation coefficient (ICC). All statistical tests were two-sided. P < .05 was considered as statistically significant. All statistical tests were performed using SPSS (v.24.0, IBM Corp. Armonk, NY).

## Results

**Fig 4:**
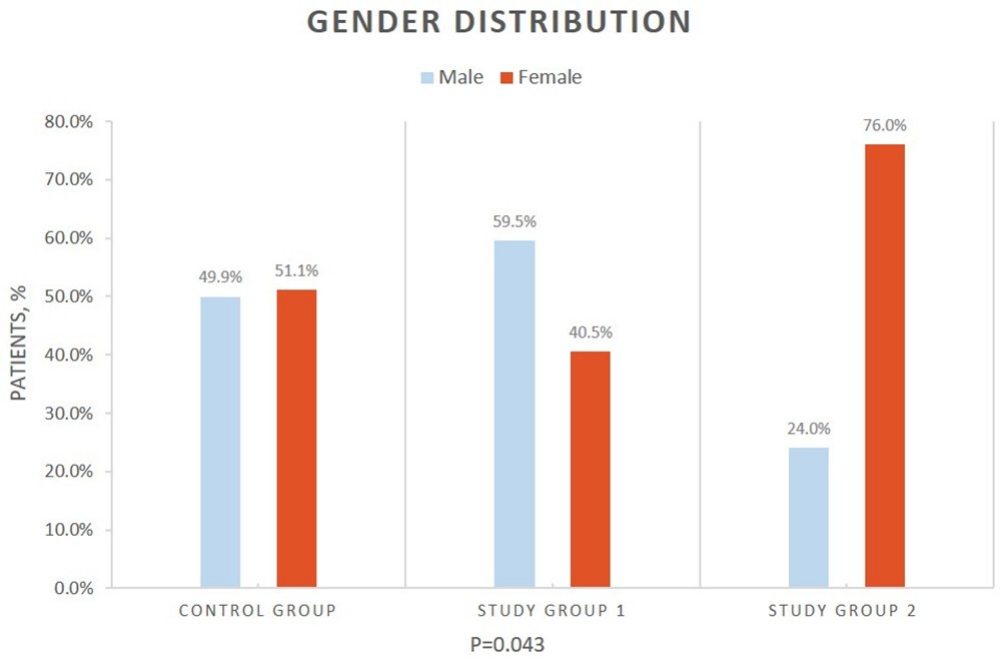
Gender distribution in study groups. A significant female predominance in patients with papilledema (study group 2) comparing to the group without papilledema (study group 1) and control group (76%, 40.5% and 51.1% respectively, p = 0.04).

After implying exclusion criteria, 153 patients with a mean age of 11.1 years (standard deviation = 5.2), 75 (49%) of whom were male, were included in the study. Table 1 and Fig 4 describe patient demographic characteristics with corresponding p-values for comparison of all clinical groups. Study group 2 (patients with papilledema) had a significant female predominance compared to study groups 1 and the control group (76%, 40.5% and 51.1%, respectively, p = 0.04).

**Table 1:** Population demographic characteristics according to study groups.

	All study population (n = 153)	Study group 1 (n = 42)	Study group 2 (n = 17)	Control group (n = 94)	p-value
**Sex** (Male/Female)	75/78	25/17	4/13	46/48	0.043
**Age** (Median IQR))	12 (7–16)	11.5(5–16)	15(13–16.5)	11(7–15)	0.014

Study group 1, Headache + No Papilledema; Study group 2, Headache + Papilledema.

The control group had a median ONSD/ETD index value of 0.17 (IQR = 0.15–0.18), study group 1 (patients with no signs of papilledema) had a median ONSD/ETD index value of 0.18 (IQR = 0.16–0.19), and study group 2 (patients with papilledema) had a median ONSD/ETD index value of 0.24 (IQR = 0.22–0.25). ONSD/ETD index was found to have significantly higher values (p<0.001) in study group 2 (patients with papilledema) compared to study group 1 (patients with no signs of papilledema) and the control group (Table 2). No correlation between ONSD/ETD index values and age was found (r = -0.086, p = 0.288).

**Table 2:** Median and IQR values of ONSD/ETD index according to clinical groups.

	Control group	Study group 1	Study group 2
**Median ONSD/ETD index**	0.17	0.18	0.24
**IQR**	0.15–0.18	0.16–0.19	0.22–0.25
**P**	<0.001		

Study group 1, Headache + No Papilledema; Study group 2, Headache + Papilledema.

**Fig 5:**
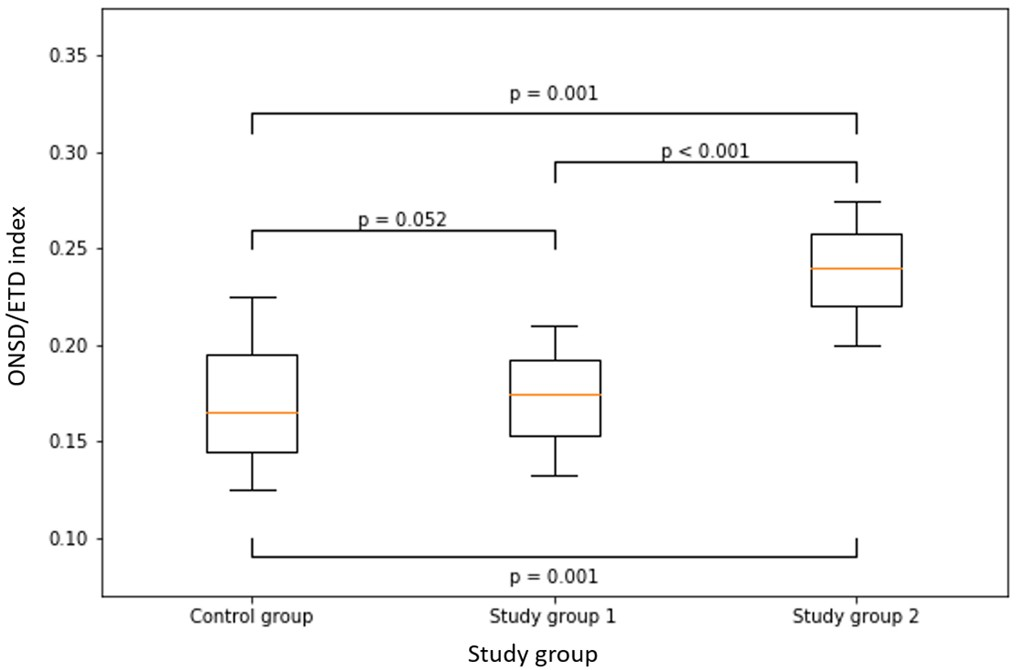
ONSD/ETD index according to clinical groups with corresponding p-values. ONSD/ETD index is significantly higher (p<0.001) in patients with papilledema (median 0.24, IQR = 0.22–0.25) compared to patients in with no signs of papilledema (median 0.18, IQR = 0.16–0.19) and control group (median 0.17, IQR = 0.15–0.18). There is no significant difference between the control group and patients with no signs of papilledema (p = 0.052).

There was no significant difference in mean ONSD/ETD value comparing study group 1 and the control group (p = 0.052). Statistically significant differences were found when comparing study group 1 (no papilledema) and study group 2 (p<0.001) and between the control group and study group 2 (p<0.001) (Fig 5). An excellent Interobserverve agreement was observed (ICC range between 0.86–0.98).

**Fig 6:**
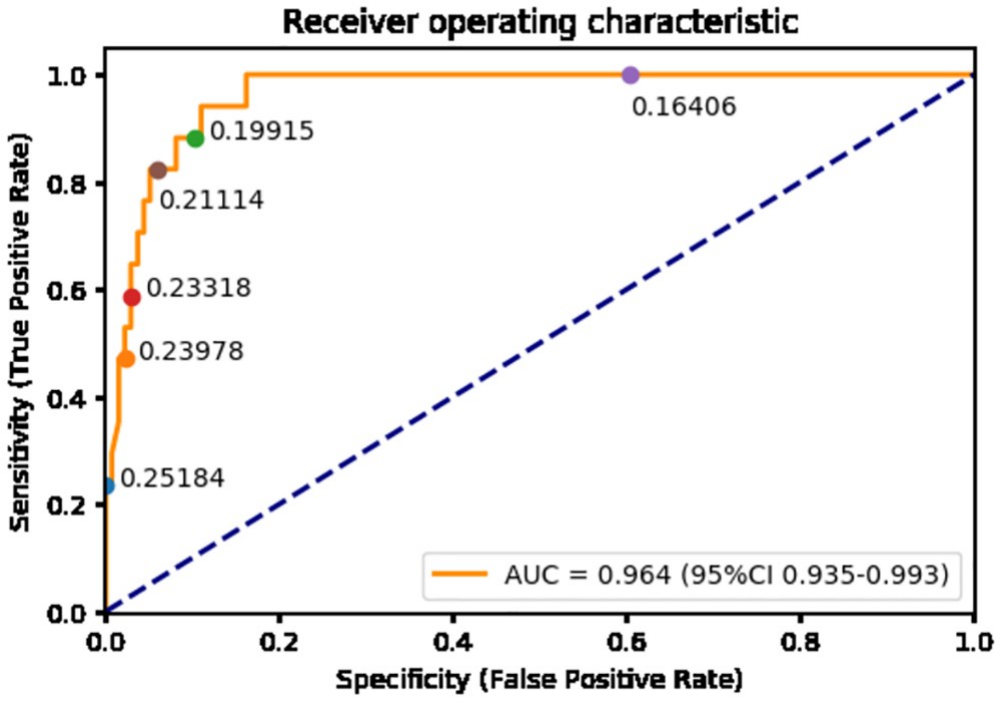
Receiver operator characteristic curve at different thresholds of ONSD/ETD index.

The ONSD/ETD index showed an excellent discrimination ability for patients with headache papilledema (AUC = 0.96, 95%CI, 0.94–0.99). An ROC curve at various thresholds of ONSD/ETD index is shown in Fig 6. The ONSD/ETD index of 0.21 was found to be 82% sensitive and 93% specific for identifying pediatric patients with headache and papilledema.

## Discussion

The current study analyzes the relationship between ONSD/ETD index values and how they relate to the presence of papilledema in pediatric patients presenting with headache due to papilledema being a well-established indicator for increased ICP and the ability of increased ICP to cause to ONSD enlargement [14,16,18]. Patients with papilledema expressed increased ONSD/ETD index values compared to those without. Additionally, an ONSD/ETD index value of 0.21 demonstrated excellent sensitivity and specificity (82% and 93%, respectively). Calculating ONSD/ETD index value can therefore be used to diagnose papilledema and, given papilledema’s acceptance as a surrogate marker of increased ICP, ONSD/ETD index values can also be used to diagnose elevated ICP. This is important given the well-known difficulty in diagnosing increased ICP in pediatric patients with headache due to unknown reason.

The lack of correlation between ONSD/ETD index values and age indicates that this is a constant ratio, which is consistent with the knowledge that both ONSD and ETD increase with age. Given this, ONSD/ETD index values can be used as universal tool for all age groups. Furthermore, it is possible that changes in ONSD/ETD index value in a single patient (assuming previous brain CT scans are available for comparison) can also be used for comparison, although further research would need to be done to confirm this hypothesis.

To the best of our knowledge, while similar studies having been performed on adults, no studies evaluating the ONSD/ETD index as a possible indicator for elevated ICP have been performed in pediatric patients. Prior studies have been conducted to try to develop the technique of using ONSD measurements in the diagnosis of increased ICP. Multiple studies were held in attempt to diagnose increased ICP in pediatric population using other different imaging modalities. Several studies conducted using measurements of ONSD by ultrasound suggested possible cut off values of ONSD diameter, with upper limit values which varied between 4 to 4.5 mm [6,19]. Shofty et al. examined the correlation between ONSD width and elevated ICP in pediatric patients by comparing the mean ONSD in idiopathic intracranial hypertension (IIH) (previously referred to as pseudotumor cerebri) cases with ONSD of healthy controls, utilizing MRI scans. The data revealed that ONSD was significantly larger in IIH patients. ONSD values were found to be positively correlated with age [15]. However, using ONSD alone has demonstrated significant variability, thus questioning the precision of ONSD in diagnosing papilledema and elevated ICP [16].

Meanwhile, Vaiman et al. hypothesized that ONSD/ETD index values may be more precise than ONSD alone, and their subsequent study conducted by Vaiman et al. showed that the middle part of the intraorbital path was the optimal site for ONSD measurements in order to calculate the ONSD/ETD index [16]. We, on the other hand, measured ONSD at a point where the ophthalmic vein crosses the optic nerve, although this site shouldn’t be affected by globe positioning, and was used in several other studies showing possible implications of ONSD/ETD index in cases of increased ICP secondary to different pathologies [20–22]. Furthermore, Vaiman et al. studied papilledema in adults, whereas we looked to see if this method of identifying papilledema applies to pediatric patients, as well [13,16,20–22].

Although MRI is the modality of choice in the neurologic workup of pediatric patients due to the lack of radiation exposure, CT scans still play a major role in the evaluation of pediatric patients with headache especially in emergency department and in countries with low MRI availability. The results of our study indicate that the measurement of ONSD and ETD based on CT scans, which allows for calculation of ONSD/ETD index, can be used as an additional diagnostic tool for the diagnosis of increased ICP, in addition to the traditional, less specific markers of increased ICP, in otherwise normal brain CT among pediatric patients with headaches. These results, obtained from CT scans, suggest that ONSD/ETD is a strong and promising diagnostic tool. However, as MRI is superior to CT in neuroimaging, and optic nerve imaging in particular, additional studies should be performed using MRI, in order to strengthen the results of the current study. Furthermore, while we established that an ONSD/ETD index value of 0.21 is both highly sensitive and specific, it is possible that MRI studies may produce a slightly different, more precise index value. Furthermore, it is also possible that MRI studies may indicate other possible signs of increased ICP. Nonetheless, the current study indicates that the ONSD/ETD ratio is a technique may aid physicians in medical decision making about additional work up and appropriate treatment.

However, it should be taken into consideration that papilledema is known to be both sensitive and specific as an increased ICP indicator in children older than 8 years. Hence, this indicator should be used in caution when evaluating younger children. The application of this technique in other clinical situations with intracranial hypertension, such as traumatic and non-traumatic brain hemorrhage should be further investigated.

The current study is not without limitations. The current study utilizes CT scans, despite the fact that MRI scans are considered superior for visualizing the optic nerves; this is due to the limited emergency department MRI availability in the country that this study was performed. Because of this, the current study may be looked at as a pilot study requiring further confirmation and validation with MRI scans. Additionally, although our study size was fairly standard (N = 153), these patients were then stratified into three groups (two study groups and a control), leaving only 42 patients in study group 1, 17 in study group 2, and 94 in the control group. Ideally, we would have liked to have larger study groups, but due to the specifications of each study group, especially study group 2, this was not possible. The data about papilledema on ophthalmological study was collected retrospectively and the examination was held by different doctors, increasing the possibility for bias. Furthermore, the definitive diagnoses of most patients in the study groups was unknown, which could have confounded our results. Additionally, majority of these patients were over the age 10, although it is consistent with the trend of increasing prevalence of headache with increasing age. Similarly, there was a female predominance in study group 2 compared to those in study group 1 or the control group. At this same time, a female patient with papilledema raises suspicion for pseudotumor cerebri, which can occur at any age but is usually only diagnosed in young adulthood. It is possible that some of these patients do in fact have this condition, in which case using ONSD/ETD ratio can potentially be used to diagnose the condition earlier. A larger cohort of pediatric patients with further follow up representing different conditions from spectrum of diseases associated with increased ICP would be needed to establish accuracy of our method as a possible tool for diagnosis of increased intracranial pressure.

## Conclusion

Our study shows that the ONSD/ETD ratio may be used for diagnosing papilledema in pediatric patients. Using this as a diagnostic tool will assist in early diagnosis of increased ICP, thereby assisting clinicians in identifying high-risk patients and allowing for further clinical workup, early intervention, and subsequent management in these patients. The current study, based on CT scans, found an ONSD/ETD index of 0.21 to have a strong balance of sensitivity and specificity (82% and 93%, respectively). However, further research should be done on MRI scans, instead of CT scans, for validation and more precise index value measurement.
